# Clinical Strategies and Therapeutics for Human Monkeypox Virus: A Revised Perspective on Recent Outbreaks

**DOI:** 10.3390/v15071533

**Published:** 2023-07-12

**Authors:** Nilanjan Ghosh, Leena Chacko, Jayalakshmi Vallamkondu, Tanmoy Banerjee, Chandrima Sarkar, Birbal Singh, Rajkumar Singh Kalra, Jasvinder Singh Bhatti, Ramesh Kandimalla, Saikat Dewanjee

**Affiliations:** 1 Molecular Pharmacology Research Laboratory, Department of Pharmaceutical Technology, Jadavpur University, Kolkata 700032, West Bengal, India; nils.bhu@gmail.com (N.G.); rhythmtanmoy@gmail.com (T.B.); 2 BioAnalytical Laboratory, Meso Scale Discovery, Rockville, MD 20850-3173, USA; leensmanoj@gmail.com; 3 National Institute of Technology, Warangal 506004, Telangana, India; vlakshmij@gmail.com; 4 Advanced Pharmacognosy Research Laboratory, Department of Pharmaceutical Technology, Jadavpur University, Kolkata 700032, West Bengal, India; chandrimasarkar321@gmail.com; 5 ICAR-Indian Veterinary Research Institute (IVRI), Regional Station, Palampur 176061, Himachal Pradesh, India; singh-birbal.singh@icar.gov.in; 6 Okinawa Institute of Science and Technology, Graduate University (OIST), Onna-son, Okinawa 904-0495, Japan; rajkumar.singh@oist.jp; 7 Laboratory of Translational Medicine and Nanotherapeutics, Department of Human Genetics and Molecular Medicine, School of Health Sciences, Central University of Punjab, Bathinda 151001, Punjab, India; jasvinder.bhatti@cup.edu.in; 8 Department of Biochemistry, Kakatiya Medical College, Warangal 506007, Telangana, India

**Keywords:** monkeypox virus (MPXV), human monkeypox (HMPX), epidemiology, MPXV pathophysiology, diagnosis, therapeutics

## Abstract

An enveloped double-stranded DNA monkeypox virus (MPXV) is a causative agent of the zoonotic viral disease, human monkeypox (HMPX). MPXV belongs to the genus Orthopoxviridae, a family of notorious smallpox viruses, and so it shares similar clinical pathophysiological features. The recent multicountry HMPX outbreak (May 2022 onwards) is recognized as an emerging global public health emergency by the World Health Organization, shunting its endemic status as opined over the past few decades. Re-emergence of HMPX raises concern to reassess the present clinical strategy and therapeutics as its outbreak evolves further. Keeping a check on these developments, here we provide insights into the HMPX epidemiology, pathophysiology, and clinical representation. Weighing on its early prevention, we reviewed the strategies that are being enrolled for HMPX diagnosis. In the line of expanded MPXV prevalence, we further reviewed its clinical management and the diverse employed preventive/therapeutic strategies, including vaccines (JYNNEOS, ACAM2000, VIGIV) and antiviral drugs/inhibitors (Tecovirimat, Cidofovir, Brincidofovir). Taken together, with a revised perspective of HMPX re-emergence, the present report summarizes new knowledge on its prevalence, pathology, and prevention strategies.

## 1. Introduction

Human monkeypox (HMPX) virus, another deadly Orthopoxvirus (OPXV) that was causing viral sickness in people at the time, became the primary priority of agencies when the World Health Organization (WHO) declared smallpox eradication in 1980 [1]. Among the known 10 species under genus OPXV, the variola (smallpox) virus had high virulence and case fatality rate (30%) and was recognized as the most deadly viral illness in humans [2]. Monkeypox virus (MPXV), another member of the OPXV group, is highly capable of propagating among mammals, including humans. The incubation period of the virus, in case of human-to-human transmission, is 12 days. It is believed that the virus spreads via respiratory secretions and saliva, or via direct contact with the exudate/crust material of the lesion. The transmission of the virus is also possible through viral shedding via fecal matter.

MPXV is an enveloped, oval-shaped, double-stranded DNA (dsDNA) virus with a size range of about 200–250 nm, resembling about 96% similarity in the genome with that of the variola virus. Understandably, the symptoms of MPXV infection are quite similar to that of smallpox, though less severe [3]. MPXV infection was first reported in the Democratic Republic of Congo in 1970; however, at present it has registered its footfall in all the continents except Antarctica [4]. The recent outbreak in 2022 has renewed concerns about HMPX. West African MPXV infections show less severity in humans, whereas Congo MPXV induces receptor-mediated activation of T-cells. The Congo clade contains a gene that inhibits complement enzymes, whereas the West African clade is devoid of this gene. This absence of an essential immune-modulating factor expression might be attributed to the enhanced virulence of the Congo clade relative to the West African clade [4].

As per the Centers for Disease Control and Prevention (CDC) guidelines, there is currently no specific treatment for MPXV infections. Antiviral drugs approved for smallpox may be used to treat HMPX. However, the clinical outcome and the risk-benefit ratio remain unclear. The WHO has already warned that the world might face another challenge resembling the COVID-19 pandemic, in the form of the HMPX outbreak. Hence, it is the need of the hour to prepare ourselves with a directory of preventive and therapeutic modalities. The present article summarizes the pathophysiology, and clinical scenario with regard to MPXV infections to guide researchers toward better management of HMPX.

## 2. Epidemiology

**Figure 1:**
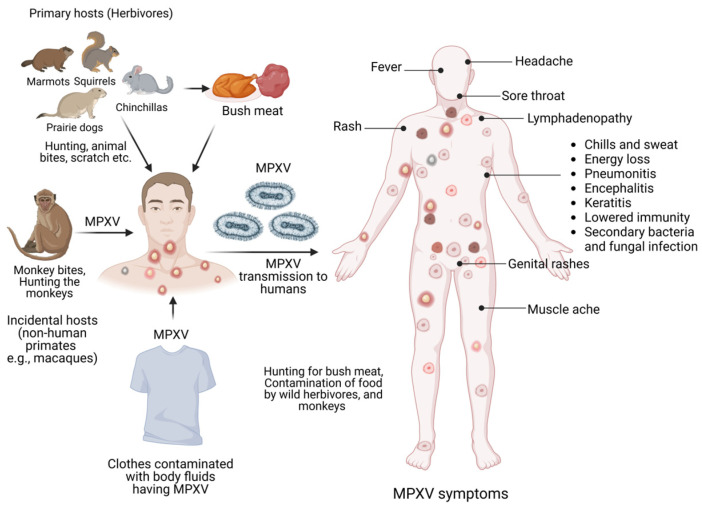
Schematic diagram showing different modalities of MPXV infection and raised pathophysiological symptoms.

The MPXV was first identified in Copenhagen, Denmark and isolated from Cynomolgus monkeys in 1958. Since then, it has been limited to African nations as a zoonotic disease (Figure 1). In 1970, the very first MPXV human infection was reported in a 9-month-old male infant in the Democratic Republic of the Congo (DRC) [4]. For the next few decades, other sporadic cases followed it from Central and West African countries like Liberia, Nigeria, and Sierra Leone.

The DRC confirmed the first MPXV epidemic in 2003 and South Sudan in 2005 [5,6]. From 2000 to 2009, three African nations documented 92 MPXV occurrences, whereas seven African nations recorded 277 MPXV cases between 2009 and 2019. In September 2017, the largest MPXV outbreak was recorded in Nigeria. In total, 1347 confirmed cases and 28,815 probable instances of MPXV were recorded in 2019 [7]. Before the present epidemic, no countries other than Central and Western Africa documented MPXV epidemiology. However, there was a sudden outbreak of 79 patients in the USA in 2003 due to the import of ill rodents from Ghana [8]. Until May 2022, 31 nations outside the regular MPXV endemic zones detected the arrival of MPXV, which proceeded to increase rapidly [9]. As a result, on June 22, 2022, the World Health Network (WHN) designated the recent MPXV outbreak as a pandemic, reporting 3417 active MPXV cases in 58 different countries [10].

A noteworthy aspect of MPXV-infected patients indicates that the epidemiology of this recent wave points towards men who had sexual activity with a male partner (MSM) and was thought to be spreading from close contact (skin-to-skin) within the close sexual networks [11]. The average age of the victims of this global pandemic was 35, and 97.4% were male. MSM was the recognized sexual preference in 97.5% of individuals, with 1% being bisexual [12]. There is a danger of communal transmission as, since the initial occasion, a huge proportion of MPXV-infected individuals have been recorded in the UK after human exposure via close contact. The usual symptoms of MPXV were absent in several individuals in this epidemic. Genital or perineal rashes are often the first sign of syphilis, suggesting that intimate physical contact during sexual intercourse is the likely vector for infection (Figure 1) [13].

## 3. Pathophysiology

**Figure 2:**
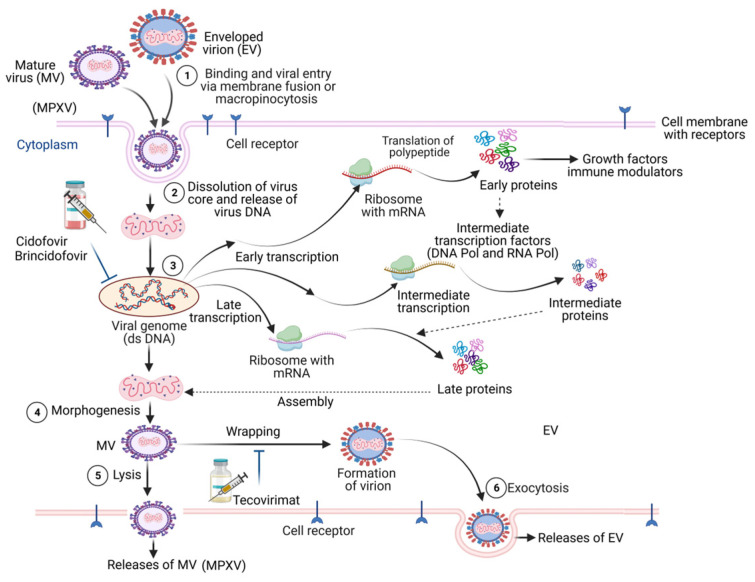
Schematic overview of the mechanism of MPVX infection in human cells. MPVX mature viruses (MV) and enveloped viruses (EV) bind and make an entry (step-1) to the cells via membrane fusion or micropinocytosis. MPXV releases its genome on arriving in the cytoplasm (step-2), where it executes transcription programs (step-3) early (synthesizing polypeptides/proteins that function as growth factors and immune modulators), intermediate (synthesizing intermediate transcription factors including DNA pol and RNA pol), and late (synthesizing proteins involved in MPVX genome assembly) stages. Antiviral medications Cidofovir and Brincidofovir target this step to inhibit MPVX infection. Assembly of the MPVX genome and core elements subsequently undergoes morphogenesis (step-4) and produces MV, which can either release from the host cell membrane directly (step-5) or may undergo wrapping to produce EV that is released by exocytosis (step-6). The later step is the therapeutic target of Tecovirimat.

MPXV is a dsDNA virus and is reported to cause monkeypox disease in humans and several other animals (Figure 2). MPXV is a member of the genus OPXV in the Poxviridae family that is known to have a large and complex DNA content amongst all animal viruses [14]. The MPXV has four elements in its virion that include the core, outer membrane, lateral bodies, and the outer envelope made of lipoproteins [15]. The dsDNA genome and fibrils are encapsulated within the core. The MPXV dsDNA genome is 197 kb in size, while the central genomic region is 101 kb. Other genomic elements consist of 6379 bp terminal inverted repetition (ITR, at both terminal variables regions) with ∼80 bp long hairpin loop, short tandem repeats (70 or 54 bp), and exclusive ITR regions NR1 and NR2 and the existing coding region [14,15,16].

The MPXV genome has ∼190 nonoverlapping open reading frames (ORFs), while four are located in the ITR sequence. As consistently seen in OPXVs, the central genomic region consists of genes that have functions in viral transcription, replication, virion assembly, and its release (Figure 2). Both ends of the MPXV genome consist of genes responsible for virulence that have functions in immune evasion, essentially by interrupting the signaling, antigen presentation and recognition, and cell death [17]. The dsDNA structure, aided by DNA polymerase 3′-5′ exonuclease activity, probably contributes to a lower frequency of genomic mutations in the MPXV. However, the 2022 variant of MPXV is reported to be different from the genomes of earlier reported MPXV by approximately 50 single-nucleotide polymorphisms (SNPs) [18]. Interestingly, these SNPs indicate increased frequency for GA > AA and TC > TT alterations [16].

Several studies have highlighted that RNA editing could speed up the variations in the MPXV genome and induce mutations [19]. The mechanisms presumed to contribute include the apolipoprotein B mRNA-editing catalytic polypeptide-like 3 (APOBEC3) enzymes [20]. A phylogenetic analysis by Wang and colleagues indicates that the MPXV-2022 strain has 46 novel consensus mutations, which comprises 24 nonsynonymous mutations as compared to the MPXV-2018 strain [21]. Further investigations may shed light on which specific mutations may help the virus evade the host immunity.

MPXV executes its replication cycle in the host cell cytoplasm. MPVX mature viruses (MV) and enveloped viruses (EV) bind and make entry via membrane fusion or micropinocytosis. Though not exactly clear, viral proteins like D8L, A27L, A34R, A26L224, and H3L were believed to have a function in cell surface binding [16]. Subsequently, MPXV releases its core into the host cytoplasm which has enzymes and key factors that instigate the transcription of viral genes [22]. Viral transcription is initiated by DNA-dependent RNA polymerase, while the translation of early (polypeptides/proteins that function as growth factors and immune modulators), intermediate (intermediate transcription factors including DNA pol and RNA pol), and late (proteins involved in MPVX genome assembly) proteins is mediated by the host cell ribosomes [23]. Assembly of viral particles produces intracellular MV that resides in the cytoplasm as mature virions and are then released as extracellular enveloped viruses (EV) at the stage of cell lysis (Figure 2) [24]. Wrapping of the MV with a Golgi-derived covering to form an EV may also occur, mediated by the VP37 protein. The EV is then released by exocytosis [25]. The later step is therapeutically targeted by the antiviral Tecovirimat.

## 4. Clinical Presentation

**Figure 3:**
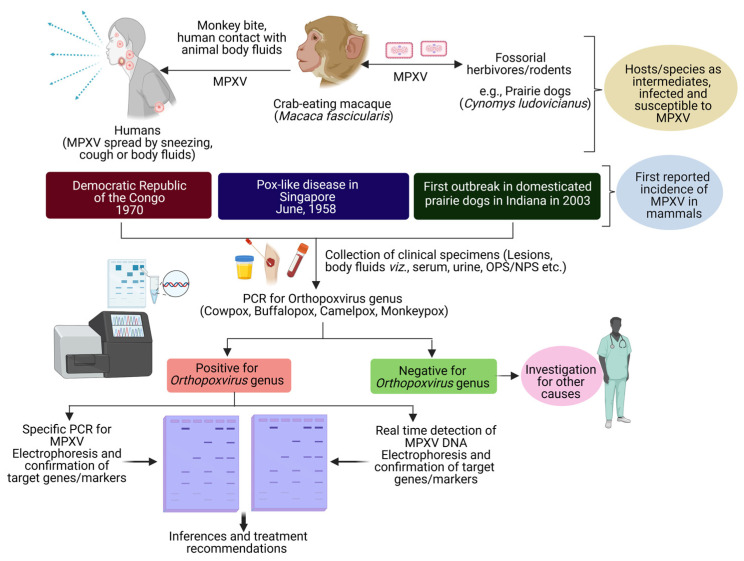
Schematic diagram showing clinical strategies enrolled for consultation and MPXV diagnosis.

Previous MPXV outbreaks were characterized by a feverish prodrome lasting from 1–4 days, followed by the development of deep, well-circumscribed cutaneous lesions, which had a centrifugal distribution [26]. Desquamation, which happened 14–24 days after the rash first appeared, was a typical last stage in the rash progression from the stages including macule, papule, vesicle, pustule, and crust. Many people with MPXV also develop lymphadenopathy [27]. MPXV typically takes around 7 days to incubate. The incubation period is when a virus multiplies and migrates from the point of injection to the lymphatic system, then to the bloodstream, and finally to other organs. Therefore, affected individuals may have symptoms for 2–5 weeks. However, during the initial 5 days of sickness, nonspecific symptoms such as fever, malaise, and sore throat might occur (Figure 3) [28].

The face rash often appears and spreads to the hands and feet. The mouth, genitalia, and eyes are all familiar places to see rashes [29]. The rash may be flat or slightly elevated, oozing clear or yellow fluid, and finally, crust over, dry up, and fall off. It is possible to tell this monkeypox from smallpox by looking for lymphadenopathy, which often appears on the groin, neck, and submandibular area during the first week of sickness. Therefore, it is essential to know how the illness manifests itself at different phases [30]. Pruritic or painful lesions, generally on the face, first appear on days 1–3. It may not manifest until day 11 of sickness, however. Circumferentially, lesions progress to the upper body, lower body, and extremities first, with the hands and feet being the last sites affected. Within 24 h of the first lesion, systemic involvement is prevalent, including the oral mucosa, genitalia, and conjunctivae [31].

Statistical evidence of lesions suggests that the face accounts for 95% of cases. Typical lesion sizes are 2–5 mm, with some reaching 1 cm in diameter. Macules are the first visible sign, followed by papules (on day 3), vesicles filled with liquid (on days 4–5), pustules with yellow fluid (on days 6–7), and ultimately crusted-over scabs (days 7–14). It has been seen that the periodic feature of the rash separates MPXV from chickenpox, where all four rash phases emerge concurrently [32].

The rash typically develops in the order outlined during the first phase of the disease, leading to the formation of lesions at different stages simultaneously. The patient is considered infectious until all scabs are gone [33]. Skin infections occur in 19% of patients, pneumonitis affects 12%, ophthalmologic problems might arise in up to 5% of patients, and encephalitis affects less than 1%. At least 10% of infected people will die during the second week of illness [34]. Several variables increase the likelihood of illness progression and severe consequences. Patients with more than 100 lesions on their skin and mucosa that cause dysphagia and, in turn, reduced oral intake and volume depletion are included in this group [35]. Infected individuals having diabetes mellitus, heart disease, and immunodeficiencies (HIV/AIDS) are at a higher risk for severe disease from smallpox than healthy children, young adults, or those who have not been immunized against the disease [36]. Genital ulcers, bigger lesions, and secondary skin infections are more common in patients with HIV, and they may persist for a more extended period [37]. The death rate is also more significant for those with HIV [29].

## 5. Diagnosis

The preliminary analysis of an individual with suspected MPXV should focus on the past medical history of the infected patient, which should include information about the patient’s journey to a location where the disease is prevalent, their exposure to infected people, and the onset of symptoms. In addition, the sexual history and whether the patient has previously received the smallpox vaccination should be noted. Health professionals should also carefully examine the oral mucosa and eyes to determine the extent of the lesions. Critically, a thorough physical examination should include testing the cardiovascular, respiratory, gastrointestinal, and nervous systems to assess the potential infection for MPXV or its exposures properly.

For the MPXV diagnosis, samples should be collected from the exudates or lesions, but ideally from an open lesion for testing. It is necessary to perform a lab test that includes total blood cell count, electrolyte levels, and a check of renal and hepatic functions. During the epidemic that occurred in 2003, aberrant results showed that 61% of patients had high levels of blood urea nitrogen and hypoalbuminemia. On the other hand, it is yet to be assessed whether the severity of the condition will increase due to these observations. The health professionals have to collect two swabs each from a minimum of three different lesions. A nylon, polyester, or Dacron swab should be used when taking samples from individuals. After that, the swab must be placed in a clean, dry container, and it needs to be stored in the freezer or refrigerator until testing. Currently, the DNA amplification check using polymerase chain reaction (PCR), or quantitative PCR (qPCR), is used most often for MPVX diagnosis (Figure 3). Although PCR has very high sensitivity and specificity, certain institutions do not have access to this technology. Alternatively, there is the option of doing immunologic serum testing to check the presence of antibodies or antigens (Figure 3). Also, there could be a chance of cross-reactivity with other OPXVs, therefore MPXV diagnosis via immunological analysis essentially lacks specificity and accuracy.

## 6. Disposition

Disposition required for patients depends on their capacity to endure oral administration, inflammation, and associated complications. Patients who cannot manage oral administration and with a high degree of algesia may require hospitalization. If the patient is stable and without complications, admission may not be required, but they should remain in strict isolation and quarantine themselves from others. Isolation is mandated until the last scabs have disappeared and a new skin layer emerges. If the patient breaks quarantine, efforts must be taken to minimize exposure to lesions and respiratory droplets.

## 7. Clinical Management

**Figure 4:**
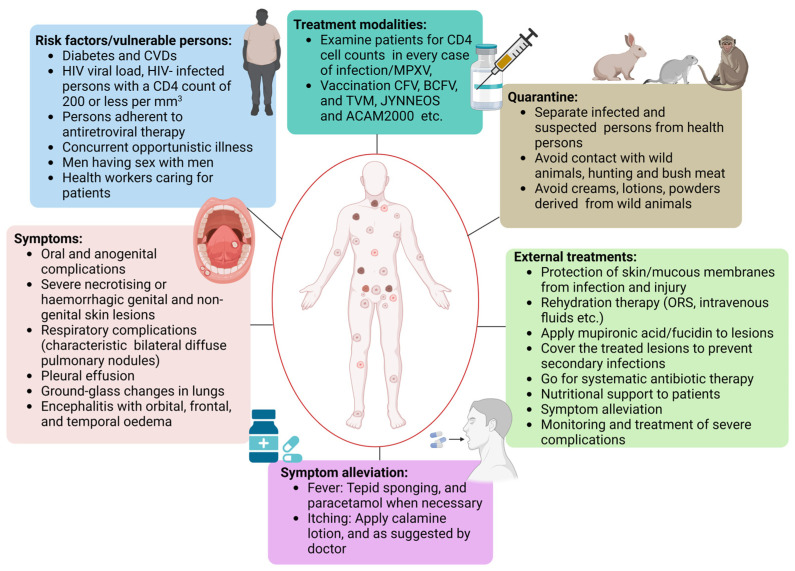
Schematic diagram showing different MPXV treatment modalities that includes consideration of patient comorbidities to calculate risk factors and review of patient symptoms. It helps health professionals to determine the course and regimes of treatment that include the vaccine, antiviral drugs, quarantine, external treatment, and careful analysis of symptom alleviation.

Presently, MPXV is only managed with supportive nursing care. The current MPXV treatment guidelines are primarily related to managing algesia, hydration, and complications (Figure 4). Hypovolemic patients may need intravenous fluid resuscitation. MPXV patients with skin infections (secondary bacterial type), conjunctivitis, or pneumonia may benefit from antibiotic treatment. Presently, MPXV infection is managed with specialized antiviral medications like Cidofovir (CFV), Brincidofovir (BCFV), and Tecovirimat (TVM). Therefore, hospitals provide nursing interventions as the core management for minor symptoms in advanced stages with impaired immune systems. A hyperimmune globulin called vaccinia immune globulin has been approved by the United States Food and Drug Administration (USFDA) for treating vaccine-related side effects in more severe situations [38]. Initially, emphasis should be on nourishment and avoiding skin lesions. External application of petroleum jelly and orally administered antihistamines are effective treatments for cutaneous lesions. Local administration of anesthetic gels, which frequently contain antihistamines, steroids, and antimicrobials, may be utilized to heal buccal infections. Local anesthetics, non-steroidal anti-inflammatory medicines (NSAIDs), and opioid analgesics could be beneficial in treating painful genital lesions in extreme situations [39].

The New York City Board of Health (NYCBOH) strain of vaccinia virus is the source material for developing the APSV vaccine [40]. Among the poxviruses, APSV vaccination is over 95% efficacious [41]. Therefore, it is anticipated that the safety profiles of ACAM2000 and APSV would be equivalent. Serious side effects of the vaccinia virus NYCBOH strain used in the smallpox vaccination are less likely to occur. However, they may still appear in adolescents who received the first dose of the vaccine. The most frequent, severe side effects after immunization are encephalitis and eczema. Similar to ACAM2000, it was predicted that APSV would increase the severity of myopericarditis. When ACAM2000 is not accessible, the USFDA has authorized APSV for usage as an emergency measure (Figure 4) [42].

Skin problems and infections brought on by the vaccinia virus are recognized as appropriate uses for the Vaccinia Immune Globulin Intravenous (VIGIV). In addition, in immunocompromised patients who might have been susceptible to MPXV and ineligible for existing smallpox vaccines, VIGIV could be used as a post-exposure precautionary agent [43]. Finally, in extreme instances of MPXV infections, VIGIV could be utilized. Unfortunately, it is unknown whether VIGIV works well for people with MPXV [44].

### 7.1. Vaccines

There is a dearth of information on how well the two currently available vaccines protect against the MPXV epidemic. Therefore, the JYNNEOS and ACAM2000 vaccines are the primary vaccines under consideration for the prevention of MPXV (Figure 4).

#### 7.1.1. JYNNEOS Vaccine

The USFDA has already approved the JYNNEOS vaccine to treat MPXV infections. The European Medicines Agency approved it in 2013 for the smallpox vaccine, and it has been marketed under the brand IMVANEX since then. Vaccinations against smallpox and MPXV are available as JYNNEOS in the United States and IMVAMUNE in Canada. IMVAMUNE was licensed in 2019 by the FDA and in Canada by 2022 [45]. The live vaccinia virus used in the JYNNEOS vaccine cannot reproduce well in human cells, making it a nonreplicating vaccination. It has shown promising results as a smallpox vaccine in animal models and clinical trials [46]. JYNNEOS is acceptable for persons living with HIV and those with exfoliative skin disorders, unlike the ACAM2000 vaccination. Immunity peaks 14 days after the second subcutaneous injection of this vaccine, which is given 28 days apart. The Centers for Disease Control and Prevention is working on a protocol for the extensive use of JYNNEOS in children. Several preclinical studies exhibited promising protective effects of JYNNEOS against MPXV [47,48]. In addition, it was found to offer excellent protection in macaques challenged with MPXV and provide long-lasting immunity [49]. Petersen and colleagues assessed the effectiveness, immunogenicity, and safety of the JYNNEOS vaccine in subjects of the democratic republic of Congo. The study is a prospective cohort of healthcare workers aged 18 years and older, in which the participants receive two doses of the vaccine on days 0 and 28 [50].

To evaluate the efficacy of post-exposure prophylaxis on the secondary transmission of MPXV, the Monkey Vax study has been launched. Adults exposed to MPXV fewer than 14 days before will be included in prospective multicenter cohort research conducted throughout the country. The primary objective of the study is to assess the failure rate of post-exposure prophylaxis vaccination with JYNNEOS within 28 days of the first vaccine dose [51].

#### 7.1.2. ACAM2000 Vaccine

Even though the USFDA officially recognizes ACAM2000 for treatment against the smallpox virus, an increased Priority Investigational New Drug application has allowed its use against MPXV. Replicating the vaccinia virus is included in the ACAM2000 vaccination [33]. This fact mandates the very cautious use of ACAM2000 in immunocompromized patients. Additionally, concerns have been raised related to the tolerability of ACAM2000, as it is involved with both myocarditis and pericarditis [52]. Some studies support the value of ACAM2000 in preventing smallpox viral infection; however, there are very little data establishing its benefit in MPXV infections.

*Cynomolgus macaques* vaccinated with ACAM2000 (single dose) were entirely protected from MPXV infection when challenged by the MPXV administered by aerosol route [53]. In a similar study evaluating the effects of ACAM2000 and TVM coadministration in nonhuman primates, all monkeys (*n* = 12) were challenged with virulent MPXV post ACAM200 vaccination; all the vaccinated monkeys fared well. Finally, in a prairie dog model evaluating the effects of ACAM2000 post-exposure, ACAM2000 exhibited significant effectiveness when administered on the first and third days of post-exposure [54].

### 7.2. Vaccinia Immune Globulin Intravenous (VIGIV)

VIGIV is a US FDA-approved medication for smallpox treatment. The vaccinia immune globulin (IgG) vaccine (VIGIV) is a sterile solution containing anti-vaccinia virus IgG antibodies titer collected from normal individuals that previously received live vaccinia virus vaccination. The CDC holds an Expanded Access Investigational New Drug Protocol (EA-IND), which permits VIGIV for MPXV treatment (https://www.cdc.gov accessed on 27 May 2023).

### 7.3. Antiviral Drugs

Treatment is necessary for MPXV patients observing severe disease and individuals that are at high risk of establishing serious diseases like the immunodeficient and the pediatric population. Individuals with dermatitis or a history of it, and pregnant or breastfeeding women also constitute a high-risk group. MPXV has no specific therapeutic regime. Many antiviral drugs, such as BCFV, TVM, and CFV, are reported to have varying degrees of effectiveness against MPXV (Figure 4) [55].

#### 7.3.1. TMV

TVM is a small molecule inhibitor of viral replication that has shown promising efficacy against OPXVs, including vaccinia, cowpox, variola, and MPXV [56]. TVM medication was initially discovered in 2002 using an in silico screening method. It has now been proven effective against several OPXVs like MPXV and ectromelia [57]. TVM inhibits the VP37 protein, which is implicated in wrapping the intracellular mature virus with a membrane and transforming the mature virus into an enveloped virus. As a result, it stops viruses from spreading in the body by impairing their release from exiting infected cells [58]. Of note, the mature virions become confined inside the host body until the cell disruption if it cannot exit the cell [59]. With an EC_50_ value between 0.01 and 0.07 μM, TVM showed potent antiviral activity [60]. In MPXV-induced animal models, TVM showed a lower fatality rate with approximately a 90% chance of survival [61]. However, preclinical results in rats receiving delayed therapy showed increased death compared to rats receiving timely doses [62]. In 2022, ST-246 received European approval for emergency use in MPXV treatment [63]. The initial day after oral TVM treatment, all skin lesions scabbed over, and mucosal lesions, which had appeared previously due to the viral invasion, had been healed (NCT05534984 and NCT05534984). In monkeys, 10 mg/kg, equivalent to 400 mg of TVM in humans, was shown to be the lowest efficacious dosage for lowering infection rates and lesions [64].

TVM has been demonstrated to possess an antiviral effect against the MPXV-2022 strain in vitro in nanomolar ranges [65]. Additionally, some in vivo studies also corroborate the effectiveness and safety of TVM [66]. Grosenbach and colleagues reported that TVM administered at 10 mg/kg b.w. for two weeks achieved more than 90% survival in the MPXV model [57]. Researchers evaluated the effectiveness of TVM against the MPXV virus in prairie dogs and reported that all the animals that received TVM survived [67]. A retrospective observational study in people infected with MPXV (7 patients) showed TVM administration at a dose of 600 mg (B.D, peroral) for 2 weeks was able to reduce the duration of illness (hospitalization for 10 days) and was well tolerated [68].

#### 7.3.2. CFV

CFV works well against almost all DNA viruses. CFV has shown to be quite efficient in treating patients suffering from vaccinia viral infection with chronic immunodeficiency. Using CFV topically and intravenously in immunosuppressed individuals has demonstrated potential efficacy against MPXV [69]. Clinical use of CFV for treating cytomegalovirus retinitis in individuals with acquired immunodeficiency syndrome (AIDS) has been authorized [69,70]. It is a prodrug, and for its activation, it needs to be phosphorylated by cellular kinases to a diphosphate derivative. This active metabolite of CFV is an inhibitor of the viral DNA polymerase and DNA polymerase 3′–5′ exonuclease functions [71]. Some in vitro and in vivo investigations point towards the benefits that CFV may offer in managing MPXV infections [72]. Similar results were reported where CFV ameliorated virus-induced cutaneous lesions in murine models [73]. It is necessary to administer intravenous saline and probenecid with CFV treatment in MPXV patients [70].

CFV reduces morbidity and the intensity of cutaneous MPXV lesions in test animals [47]. CFV treatment in mice exposed to the cowpox virus effectively suppressed viral loads and interleukin (IL)-10, IL-2, IL-3, and IL-6 [74]. In a study conducted on cynomolgus monkeys with MPXV infection, CFV treatment resulted in lessened mortality and minimized numbers of cutaneous lesions [47]. Few reports have highlighted the success of CDV against the poxvirus. Treatment of CFV dramatically improved the health of individuals with resistant *Molluscum contagiosum* virus [75].

#### 7.3.3. BCFV

BCFV is the prodrug of CFV, which was approved in 2021 for managing smallpox. BCFV is believed to have superior efficacy and tolerability compared to CFV [76]. BCFV is a lipid conjugate of CFV, which, after it enters target cells, is cleaved by phospholipase. This leads to the release of CFV, which is subsequently activated by two sequential phosphorylations, and CFV diphosphate is formed, which is responsible for the antiviral activity [77]. Compared with CFV, BCFV has better antiviral activity due to elevated intracellular penetration of the active drug and superior oral bioavailability. Additionally, the tolerability was better, with a lower incidence of nephrotoxicity [78]. While evaluating an MPXV animal model comprised of STAT1-deficient C57BL/6 mice, treatment of BCFV and TVM successfully protected these mice from MPXV. The combination of BCFV and TVM was started on infection day [79].

The pharmacokinetic parameters following oral administration of BCFV were determined using the prairie dog MPXV model. BCFV exhibited beneficial effects, and its efficacy was observed to be dependent upon the onset of treatment; the sooner therapy began, the better the prognosis [80]. To determine if the magnitude of the infectious virus dosage is related to the efficacy of BCFV treatment, researchers employed the murine model of smallpox. As early as day 3 or 4 post-exposure, ectromelia virus viral DNA has been detected in the oral swabs of mice across various dosages, prompting the beginning of therapy. The findings showed that the therapeutic window for BCFV therapy diminishes with increasing viral infectious dosage [81].

#### 7.3.4. Antiviral Drugs: Real-Life Experiences and Toxicities

Although these (TMV, CFV, BCFV) antiviral drugs, given their antiviral efficacies, are presently being enrolled as preferred treatments for MPVX, patients’ real-life experiences have varied. The phase I and II studies of TMV have shown that it is safe and well tolerated [57]. TMV is often solubilized with B-cyclodextrin prior to administration due to its lesser water solubility that possesses no significant toxicity, as examined in patients with renal impairment [82,83]. CFV was shown to promote dose-limiting nephrotoxicity that exhibits clinical features of proteinuria and glucosuria, and declined bicarbonate, phosphate, and uric acid. Continued CFV medication was further seen to increase serum creatinine levels, which could lead to severe nephrotoxicity [84,85,86]. Mechanistically, dose-dependent CFV severity was caused by the action of organic anion transporter 1 (OAT1) that promotes its accumulation in the kidney proximal tubule cells [73]. The phase I/II studies in AIDS patients revealed prehydration and decreased probenecid rate of nephrotoxicity, specifically in patients given CDV doses of more than 3 mg/kg. Considering these issues, CFV medication is avoided in patients that have greater serum creatinine levels and proteinuria conditions [86]. In the case of BCFV, phase I/II/III studies revealed dose and frequency-associated hepatocellular and gastrointestinal toxicity as a common adverse effect [87]. BCFV had the advantage of oral administration and showed a lower degree of nephrotoxicity as compared to CFV as well [87].

## 8. Conclusions

The outbreak and prevalence of MPXV in many countries that were not endemic to MPXV infection exhibited no traveling history of infected cases to the endemic areas. Although MSM sexual activities have been central in the recent 2022 HMPX outbreak, spreading controlling, vigilant activities, including surveillance, contact tracing, and community-centric initiatives, especially in viral hotspots regions, should be promoted as a priority. Also, promoting MPXV knowledge and awareness is the key to preparedness and effective management, especially among healthcare workers and professionals. On this front, several lessons can be learned from the COVID-19 pandemic. Of note, present evidence points to MSM activities as an underlying cause, though infected cases were not exclusive to this cluster. Therefore, these preliminary data must be meticulously analyzed and interpreted to provide clear information and avoid ambiguity, which is also essential to avoid any potential bias towards infected MSM cases.

With a revised perspective of HMPX re-emergence, the present report attempted to gather new knowledge on HMPX prevalence, pathology, and prevention strategies. More investigation on HMPX is expected to improve our knowledge of factors involved in promoting the spread of HMPX that may enable early diagnosis and help break the transmission chain. Also, revising public health preparedness and building effective therapeutic regimes against HMPX are equally essential to contain future viral outbreaks. Advanced tools analyzing viral epitopes and structural-functional pathogenic mechanisms have accelerated the discovery of vaccines, drugs, and specific inhibitors blocking viral antigens/proteins. These drug discovery approaches have been promising; however, their safety and efficacy are critical to analyzing in systematic preclinical trials. In conclusion, it is crucial to uphold the investigative and in-depth studies in HMPX research that can elucidate the mechanism of host–viral interaction and enable accelerated drug discovery against HMPX or any potential viral outbreak in the future.

## Data Availability

Not applicable.
